# Replacing fish oil with *Tetraselmis chui* microalgae biomass does not compromise rainbow trout health: Biochemical, histologic, antioxidant and immune gene expression

**DOI:** 10.1038/s41598-026-54873-7

**Published:** 2026-06-14

**Authors:** Stanley Iheanacho, Anna Simon, Jonas Mueller, Sebastian Lippemer, Alexander Rebl, Mario Hasler, Carsten Schulz

**Affiliations:** 1 Dept. Marine Aquaculture, Institute of Animal Breeding and Husbandry, Christian-Albrechts-University of Kiel, Olshausenstraße 40, 24098 Kiel, Germany; 2 Fraunhofer IMTE, Fraunhofer Research Institution for Individualized and Cell-Based Medical Engineering, Aquaculture and Aquatic Resources, Hafentörn 3, 25761 Büsum, Germany; 3 Blue BioTech GmbH, Hafentörn 3, 25761 Büsum, Germany; 4 Research Institute for Farm Animal Biology (FBN), 18196 Dummerstorf, Germany; 5 Lehrfach Variationsstatistik, Christian-Albrechts-University of Kiel, Olshausenstraße 40, 24098 Kiel, Germany

**Keywords:** Blood markers, Fish oil, Microalgae (Tetraselmis Chui), Oncorhynchus mykiss, Physiology, Sustainable aquafeed, Biotechnology, Ecology, Ecology, Physiology, Zoology

## Abstract

Microalgae offer a nutritionally robust alternative to fishmeal and fish oil, helping reduce pressure on wild stocks and supporting more sustainable aquafeed production. This study explored the potential of replacing fish oil with Tetraselmis (*Tetraselmis chui*) microalgae biomass in the diet of juvenile rainbow trout (89.0 ± 1.10 g) (*Oncorhynchus mykiss*), assessing its effects on the fish’s health. A control diet containing 53% crude protein and fish oil (FO) was modified by replacing FO with Tetraselmis at three graded inclusion levels: 33% (Tetra33), 66% (Tetra66), and 100% (Tetra100). The 84-day feeding trial evaluated key growth parameters, biochemistry, liver and intestinal histo-architectures, and immune-antioxidant gene expression profiles of the experimental fish. Time-series analyses of growth performance revealed no significant treatment effects from day 14 to day 70, except at the 84-day biomass sampling. The FO (7321.65 ± 60.03g) attained a significantly greater final weight (FW) than Tetra33 (6984.70 ± 86.15g) and Tetra100 (6823.93 ± 160.42g), while remaining statistically similar to Tetra66 (7051.77 ± 107.30g). Likewise, weight gain (WG) of the FO group (5519.65 ± 57.16g) exceeded that of the Tetra100 group (5043.93 ± 142.09g) but did not differ significantly from the Tetra33 (5220.70 ± 73.95g) and Tetra66 (5281.77 ± 110.86g). The feed conversion ratios (FCRs) and specific growth rates (SGRs) of the Tetra groups were comparable to the FO control. Dietary variation did not elicit significant changes in leukocyte distribution, biochemical indices, or gene expression patterns across Tetra groups relative to the FO. Similarly, the histological analysis revealed that Tetraselmis dietary inclusions did not trigger inflammatory reactions in hepatic or intestinal tissues in the Tetra groups compared to the FO. Minor but inconsequential histological modifications were noted, such as moderated sinusoid dilation in the liver and slight changes in intestinal villi of Tetra33 fish. Health biomarker analyses indicated that replacing fish oil with *Tetraselmis* preserved physiological homeostasis, whereas 66% replacement (Tetra66) yielded the best growth performance compared to FO. However, longer feeding trials are necessary to confirm long-term health and nutritional outcomes.

## Introduction

Alternative feed ingredients are becoming popular in aquaculture, given their nutritional potential to replace expensive conventional feedstuffs, including fish oil. The increasing demand for fish oil by pharmaceutical, cosmetic, and food industries^1,2^ puts greater pressure on its availability for aquafeed production. Majluf et al.^3^ emphasised the rising cost of fishmeal/fish oil (FMFO), which has led to a considerable decrease in their utilisation within aquaculture, with the inclusion rate falling from 23% to 8% over the past two decades. Therefore, transitioning to low-cost feed ingredients to replace fish oil in fulfilling critical dietary requirements for aquaculture species remains important for sustainable aquaculture^4–9^. Additionally, this strategic approach will alleviate the overwhelming pressure on wild-caught fish and lessen greenhouse gas (GHG) emissions^10–12^. Fish oil provides a substantial dietary supply of essential fatty acids, including omega-3 long-chain polyunsaturated fatty acids (LC-PUFAs), which are required for promoting optimal fish growth and physiological functions^13–15^.

Microalgae, as a significant source of essential fatty acids, are recognised as a sustainable alternative to fish oil in aquaculture diets^16,17^. The use of microalgae in aquaculture feeds offers considerable potential,however, cost remains a significant constraint, influenced by factors such as substrate selection, production techniques, and the microalgal species cultivated^18^. Importantly, the cultivation approach is a key determinant in achieving nutritional enrichment of microalgal biomass, as substrate composition and culture conditions directly influence its nutrient profile^19^. Several microalga species are being explored for their significant contribution of essential fatty acids, including long-chain polyunsaturated fatty acids (PUFA), crucial for optimal fish growth, vitality, and overall health^14,20–22^. Research findings from earlier studies indicate that dietary inclusion of *Schizochytrium sp*. microalgae increased growth, digestibility, improved the gut microbiome diversity^17^ and nutritional quality of Nile tilapia (*Oreochromis niloticus*)^8^. Improved growth performance and carcass quality were reported in other fish species, such as channel catfish (*Ictalurus punctatus*)^23^ and Atlantic salmon (*Salmo salar*)^24^. Further, microalgae diets have been reported to enhance antioxidant capacity^25–29^ and the immune system of several aquaculture fish species^29,27^^,^^30^.

In freshwater, some species, including rainbow trout, possess partial biosynthetic capacity to transform n‑3 short-chain-PUFA, specifically α‑linolenic acid (ALA), into n‑3 long-chain-PUFA through a series of enzymatic desaturation and elongation reactions, yielding stearidonic acid (SDA) as an intermediate and subsequently eicosapentaenoic acid (EPA) and docosahexaenoic acid (DHA)^22,31^. This will support the adoption of novel functional feeds such as Tetraselmis microalgae with limited n‑3 long‑chain PUFA content and lower the cost burden of feed formulation. Whereas Simon et al.^13^ examined the effects of replacing fish oil with Tetraselmis on growth performance and fatty acid metabolism in rainbow trout, this present study instead examined its impact on fish health by assessing biochemical markers, leukocyte profiles, tissue histology, and gene expression profiles. Biochemical indices are critical markers for evaluating the physiological status of fish, offering valuable insights into how real-time exposures influence metabolic processes and homeostatic regulation in experimental animals^32,33^. Leukocytes are essential cellular markers for evaluating immune competence and monitoring immunological status in animals^34^. Leukocytes, acting in distinct populations, work together to protect and provide both innate and adaptive immune responses in animals. Histological assessment provides valuable insights into biological endpoints and reveals tissue structural changes resulting from dietary interventions^35^. Transcriptome expression markers are essential in providing molecular insights by revealing how specific gene regulation patterns are influenced by distinct exposures^36^^,^^37^. Collectively, these marker measurements will establish a physiological endpoint for evaluating the health effects of replacing fish oil with Tetraselmis in the diet of rainbow trout.

In the present study, Tetraselmis substituted fish oil, the primary source of n‑3 PUFA in rainbow trout’s diet. Although Tetraselmis provides no dietary DHA, rainbow trout can compensate through their endogenous pathways for DHA biosynthesis^31,38^, supporting the feasibility of this substitution. This study is also a follow-up to the previous findings reported by Simon et al.^13^, which demonstrated that replacing fish oil with Tetraselmis microalgae and plant-based oils influences fatty acid metabolism in rainbow trout, highlighting the species’ capacity to biosynthesise DHA when dietary supplies are limited. The authors also noted that several fatty acids, specifically 14:1 n-5, 21:0, 20:3 n-3, 20:3 n-6, 22:2 n-6, 22:4 n-6, and 22:5 n-6, were absent from the experimental diets yet present in whole-body samples, indicating endogenous synthesis or metabolic elongation/desaturation. While the nutritional potential of Tetraselmis microalgae as a functional ingredient in aquaculture physiology remains underexplored, this study investigated whether partially or completely replacing fish oil with Tetraselmis would influence physiological or molecular responses in rainbow trout, under the hypothesis that no adverse health outcomes would occur.

## Materials and methods

### Animal maintenance

The feeding trial was conducted at the Fraunhofer Research Institution for Individualised and Cell-Based Medical Engineering (Büsum, Germany). Female juvenile rainbow trout were procured from Forellenzucht Trostadt GmbH & Co. KG (Trostadt, Germany) and randomly distributed in 12 tanks (150 L) in a recirculating system (6.30 m^3^ total water volume, water circulation of 6 L min^−1^). The fish were acclimated to optimal rearing conditions for 2 weeks and fed a commercial diet (Aller Aqua Group, Christiansfeld, Denmark) once daily. Water quality conditions were maintained at 14.5 ± 0.6 °C temperature; 9.80 ± 0.60 mgL^−1^ dissolved oxygen; 7.24 ± 0.24 pH; 5.00 ± 1.00 PSU salinity (HI 96822 Seawater Refractometer, Hanna Instruments Inc., Woonsocket, USA), 0.72 ± 0.26 mgL^-1^ ammonia; 2.14 ± 1.74 mgL^-1^ nitrogen oxide (MQuant MColortest kits, Merck KGaA, Darmstadt, Germany). Aeration was provided to maintain water quality using an aeration compressor (Medo Kompressor LA-80B, Nitto Kohki Europe GmbH, Steinenbronn, Germany).

### Ethical approval

The guidelines of EU Directive 2010/63/EU for animal experiments and the national regulations for animal welfare (TierSchVersV) were followed, and the experiment was approved by the Ministry of Agriculture, Rural Areas, European Affairs and Consumer Protection (MLLEV, Kiel, Germany; project number IX552-27467/2024).

### Microalgae (*Tetraselmis chui*) and diet formulation

Tetraselmis was cultivated by BlueBioTech (BBT, Büsum, Germany) in airlift reactors. Cultivation conditions included a modified F-medium, a salinity of 32 PSU, a temperature range of 23-25 °C, and a pH of 8.50. After harvesting, achieved by flow-through centrifugation, the microalgae were freeze-dried using an Alpha 1-4 LSC system (Martin Christ Gefriertrocknungsanlagen GmbH, Ostrode, Germany). Subsequent homogenization of microalgae and other feed ingredients was performed with a GM 200 knife mill (Retsch GmbH, Haan, Germany) operated in reverse mode. The experimental diets for rainbow trout were formulated to meet their dietary requirements^40^ and were isonitrogenous, isoenergetic, and isolipidic (Tables 1 and 2). The reference diet (control) contained fish oil as the main n-3 PUFA (FO) source. Further, the FO was substituted with Tetraselmis at 33 % (Tetra33), 66 % (Tetra66), and 100 % (Tetra100), respectively (Table 1). Diets with incremental replacement of fish oil by Tetraselmis were used to determine whether the apparent algal lipid profile can sustain essential fatty acid supply, support metabolic performance, and maintain immune and physiological function compared with a conventional fish-oil diet. It is important to state that due to the different lipid contents of FO and Tetra, we balanced the diets with wheat starch, gluten and palm fat, as we substituted the n-3 fatty acid portion of FO by microalgae. To eliminate confounding effects from the naturally high n‑3 PUFA levels in fish meal, it was excluded from the control and test diets. Instead, poultry and plant-derived products were incorporated as protein sources to avoid experimental bias and enable accurate assessment of Tetraselmis (with low DHA content) suitability as a fish oil substitute, consistent with the study rationale presented in the introduction. Nonetheless, the control diet satisfied the experimental fish’s protein (45-55%), EPA and DHA requirements (0.7-1.0)^39–42^. The dry feed ingredients were thoroughly mixed before the oil components were incorporated. Water was added to activate the gelatin and binder during processing, which helped stabilise the pellets. All diets were pelleted using an L 14-175 press (Amandus Kahl, Hamburg, Germany), yielding 4 mm diameter pellets at a processing temperature below 60 °C. Post-pelleting, the diets underwent air-drying for 48 hours at ambient temperature before storage at 4 °C.

**Table 1:** Formulation of experimental diets.

**Ingredients (% inclusion in DM)**	**FO (control)**	**Tetra33 (33%)**	**Tetra66 (66%)**	**Tetra100 (100%)**
Soybean concentrate^*d*^	14.5	14.5	14.5	14.5
Pea protein isolate^*b*^	14.5	14.5	14.5	14.5
Poultry blood meal^*a*^	5.5	5.5	5.5	5.5
Poultry meal^*a*^	13	13	13	13
Gelatin^*c*^	3	3	3	3
L-lysin^*e*^	0.25	0.25	0.25	0.25
Pellet binder^*f*^	0.5	0.5	0.5	0.5
Vitamin premix^*g*^	0.5	0.5	0.5	0.5
CaHPO4^*h*^	1	1	1	1
Rape seed oil^*i*^	2.5	2.5	2.5	2.5
Microalga meal (*T.chui*)^*j*^	**—**	**4.67**	**9.33**	**14**
Fish oil^*k*^	**2**	**1.33**	**0.67**	**—**
Wheat gluten^*l*^	15.4	14.2	13.05	11.5
Wheat starch^*l*^	18	15.4	13.77	10.5
Cellulose^*m*^	2.3	1.65	0.25	0.25
Palm fat^*n*^	3.45	4	4.23	5.1
Linseed oil^*o*^	2.6	2.5	2.45	2.4
Titanium oxide	1	1	1	1

^a^GEPRO Geflügel-Protein Vertriebsgesellschaft mbH & Co. KG, Diepholz, Germany; ^b^Euroduna Food Ingredients GmbH, Barmc stedt, Germany; ^c^hewico GmbH & Co. KG, Nordhorn, Germany; ^d^HP 310, Hamlet Protein A/S, Horsens, Denmark; ^e^S3 Chemicals, Bad Oeynhausen, Germany; ^f^Mastercube Advanced, Anpario, Nottinghamshire, United Kingdom; ^g^Spezialfutter Neuruppin GmbH & Co. KG, Neuruppin, Germany; ^h^Lehmann & Voss & Co. KG, Hamburg, Germany; ^i^EDEKA Zentrale Stiftung & Co. KG, Hamburg, Germany; ^j^Blue Biotech Büsum GmbH; ^k^Bioceval GmbH & Co. KG, Cuxhaven, Germany; ^l^Kröner-Stärke GmbH, Ibbenbüren, Germany; ^m^Alba-Fibre C-200, Mikro-Technik GmbH & Co. KG, Bürgstadt, Germany; ^n^DF 1680 WB, Elbe Fetthandel GmbH, Geesthacht, Germany; ^o^Makana Produktion und Vertrieb GmbH, Offenbach an der Queich, Germany; NfE (nitrogen-free extract)=100 - (crude protein+crude lipid+crude ash).

**Table 2:** Nutrient compositions and fatty acids (mg/g DM) of experimental diets and *Tetraselmis chui*.

**Parameters**	**FO (control)**	**Tetra33 (33%)**	**Tetra66 (66%)**	**Tetra100 (100%)**	***Tetraselmis chui***
**Proximate content (g/100g DM)**					
Crude protein	53.88	54.40	54.75	55.30	28.61
Crude Lipid	15.45	15.46	15.10	15.49	7.39
Crude ash	5.81	6.94	8.07	8.97	24.85
Moisture	12.72	15.38	13.65	12.76	4.90
NFE	24.86	23.20	22.08	20.25	39.15
Energy (MJ kg-1)	23.11	23.07	22.89	22.96	17.63
**Fatty acids (mg/g DM) profiles**					
18:2 n-6 LA	26.58	23.40	24.20	23.73	4.07
18:3 n-6	0	0	0.13	0.17	0.99
20:2 n-6	0.19	0.15	0	0	0.09
20:3 n-6	0	0	0	0	0
20:4 n-6 ARA	0.47	0.41	0.47	0.46	0.63
22:5 n-6	0	0	0	0	0
18:3 n-3 ALA	17.19	14.89	16.21	16.62	11.25
18:4 n-3 SDA	0.38	0.45	0.59	0.74	5.04
20:3 n-3	0	0	0	0	0
20:4 n-3	0.12	0	0	0	0.11
20:5 n-3 EPA	0.96	0.76	0.70	0.60	4.12
22:5 n-3	0.25	0.16	0.13	0	0.06
22:6 n-3 DHA	1.57	0.89	0.52	0	0
**∑ PUFA**^**a**^	**48.46**	**42.07**	**43.77**	**43.22**	**27.24**
16:1	1.72	1.43	1.29	1.07	0.87
18:1 n-7	2.13	2.28	2.71	3.07	0.73
18:1 n-9	36.09	32.26	33.24	32.78	7.40
20:1	1.90	1.37	1.10	0.69	1.07
22:1 n-9	0.21	0.16	0.14	0	0
22:1 n-11	1.43	0.89	0.46	0	0
24:1	0.14	0	0	0	0
**∑ MUFA**^**b**^	**44.11**	**38.88**	**39.37**	**38.06**	**20.19**
12:0	0.20	0.21	0.20	0.21	0
14:0	1.37	1.18	0.97	0.82	0.33
15:0	0.00	0.15	0	0	0.07
16:0	43.42	46.32	49.91	57.54	12.83
17:0	0.25	0.23	0.22	0.22	0.45
18:0	4.95	4.69	4.63	4.53	0.18
20:0	0.35	0.35	0.31	0.30	0
21:0	0	0	0	0	0
22:0	0.23	0.24	0.23	0.19	0
24:0	0.14	0.14	0.14	0.12	0.1
**∑ SFA**^**c**^	**50.98**	**53.53**	**56.74**	**63.96**	**13.99**
**∑ n-3**	**20.51**	**17.25**	**18.30**	**18.11**	**21.45**
**∑ n-6**	**27.50**	**24.22**	**25.13**	**24.65**	**5.78**
**n-3/n-6**	**0.75**	**0.71**	**0.73**	**0.73**	**3.71**

a PUFA: polyunsaturated fatty acids; b MUFA: monounsaturated fatty acids; c SFA: saturated fatty acids.

### Chemical analysis

The test diets and microalgae were analysed for their proximate and fatty acid compositions (Table 2), following the European Commission Regulation No. 152/2009^43^. Each sample was measured in duplicate. Samples were dried at 103 °C for 4 hours (ED 53, Binder GmbH, Tuttlingen, Germany) to determine dry matter. Subsequently, they were combusted at 550 °C for 12 hours (P300, Nabertherm, Lilienthal, Germany) to measure ash content. Crude protein content was determined using Kjeldahl methods (KjelDigester K-449 & KjelFlex K-360, BÜCHI Labortechnik GmbH, Essen, Germany) with the conventional nitrogen-to-protein conversion factor (N × 6.25). In addition, a general microalgae conversion factor (N × 4.78) was used to estimate the crude protein content of microalgae meal, following Lourenço et al.^44^. The Soxhlet method was used to determine crude fat content, using hydrochloric acid for hydrolysis and petroleum ether for extraction (Hydrotherm & Soxtherm, C. Gerhardt GmbH & Co. KG, Königswinter, Germany). Gross energy was determined using an IKA C200 combustion calorimeter. Nitrogen-free extracts (NFE) were calculated by subtracting crude protein, crude fat, and crude ash from the total dry matter (100 - (crude protein + crude fat + crude ash)). Following the standards DIN EN ISO 11885 (E 22) and modified DGF C-VI 11a:2016 + DGF C-VI 10a:2016, fatty acids in microalgae meal and diets were analysed by AGROLAB LUFA GmbH (Kiel, Germany). Concentrations below the 50 mg/kg detection limit were reported as absent.

### Experimental trial and biomass sampling

A total of 240 female juvenile rainbow trout, with an average weight of 89.0 ± 1.10 g, were weighed in batches before being randomly allocated to four main groups (FO, Tetra33, Tetra66, and Tetra100). Female specimens were chosen exclusively due to their delayed sexual maturation and more rapid growth to the desired market size^45^. To ensure effective experimental design, each main group was further randomised into three replicate tanks, housing 20 fish per tank, adopting the Completely Randomised Design (CRD) pattern. During the 2-day post-acclimation period, the fish were starved to empty their stomach and adapt the fish to the test diets. Afterwards, the fish were manually fed the test diets daily at 1.9% of their body biomass for 84 days. Bulk tank weights (*n* = 20) were recorded at the trial’s onset, every 14 days, and at termination to enable accurate assessment of growth parameters and subsequent adjustment of daily feed allocations.

### Growth metrics

Critical growth indices such as final weight (FW), weight gain (WG), specific growth rate (SGR), and feed conversion ratio (FCR) were measured every 14-day intervals and reported using the following formula;

$${\text{Mean weight gain }} = (W_{f} {-\!\!-}W_{i} )/N$$

Where *Wf* is the final weight of fish (g), *Wi* is the initial weight of fish (g), and *N* is the number of fish

$${\text{Specific growth rate }} = \, (InW_{f} {-\!\!-}InW_{i} /T) \times {1}00$$

Where In is the logarithm; *Wf* is the final weight of fish (g), *Wi* is the initial weight of fish (g), and T is the time (days)

Feed conversion ratio: Total weight of feed consumed (g) / total weight gain of fish (g).

### Blood and tissue sampling

At the end of the 84-day feeding trial, blood, liver, and intestinal tissues were aseptically sampled under controlled hygienic conditions. Fish were starved for 48 hours before final sampling. Fish (*n* = 15, per group) were euthanised with an overdose of clove oil (1.0 mL per 10 L water) for blood analysis before tissue collection. Blood (up to 2.5 mL) was collected via caudal puncture. Blood samples were transferred into heparinised and plain tubes for subsequent haematological (leukocyte differentials) and biochemical (serum analysis) assays, respectively. Further, fish liver and intestinal tissues (*n* = 9, per group) were carefully excised and preserved in phosphate-buffered formalin for onward histological examination. A portion of the liver (*n* = 15, per group) sample was immediately placed in RNAlater and stored on ice during sampling. These samples were then incubated at 4°C overnight and subsequently stored at -20°C.

### Plasma chemistry assay

Blood samples in plain tubes were placed into a centrifuge (Heraeus Multifuge X3R, Germany) for serum collection. They were then centrifuged at 3000 RPM for 8 minutes at 4°C. The supernatant (serum) was carefully aspirated with a Pasteur pipette, transferred into plain plastic tubes, and stored at _~_ 80°C until biochemical analysis. Biochemical parameters, including glucose, total protein, cholesterol, triglycerides, amylase, lipase, bilirubin, creatinine, albumin, and alkaline phosphatase (ALP), aspartate aminotransferase (AST), and alanine aminotransferase (ALT), were determined using a multi-test automated Drychem analyser (Fuji DRI-CHEM NX500, Germany). Analytical procedures adhered strictly to the manufacturer’s guidelines

### Leukocyte differential assay

The leukocyte count was determined after diluting the blood 1:20 (v/v) with white blood cell (WBC) diluting fluid, as per the procedure outlined by Hesser^46^. Blood (0.02 ml) was drawn to the 0.5 mark on a white cell pipette, then pipetted into a small test tube where 0.38 ml of dilution fluid was added. A few drops of the diluted blood were introduced into the hemocytometer. A differential leukocyte count was done by identifying 200 consecutive leukocytes using a 40× objective. Afterwards, each specific leukocyte cell was divided by the total number of leukocytes counted, and then multiplied by 100, to obtain their respective percentages.

### Histological assay

Liver and intestinal tissues (*n=*9 per group) were dehydrated, paraffin-embedded, sliced into 4 μm sections, and stained with hematoxylin and eosin using routine histopathological surgical methods^47^. Samples were randomly examined using a light microscope (Primo Star, Carl Zeiss Microscopy GmbH). The pertinent tissue sections were examined to discern microalgae-induced modifications in tissue histoarchitecture. The following changes were evaluated in the fish liver, including intrahepatic erythrocytes, distended central vein, sinusoidal dialation, and vacuolation (Merrifield, 2011). Histological observations were quantified using a five-point severity grading scale (0-5)^48^. Scores were blindly assigned as follows: 0 (no observation), 1(normal), 2 (mild), 3 (moderate), 4 (severe), and 5 (very severe).

### RNA extraction and gene expression

The fish liver (*n* = 15, per group) was sampled for ribonucleic acid (RNA) isolation. Total RNA was extracted using TRIzol (Thermo Fisher Scientific, Waltham, USA). Subsequently, the RNA was purified with the ISOLATE II Mini Kit (Meridian Bioscience Inc., Cincinnati, Ohio, USA). Isolated RNA concentration and quality were measured using a NanoDrop One^C^ (Thermo Fisher Scientific). High-quality RNA was then reverse transcribed into cDNA using the Reverse Transcription Master Mix (Standard BioTools, South San Francisco, California, USA). Subsequently, cDNA samples were pre-amplified using the Fluidigm PreAmp Master Mix and treated with Exonuclease I (New England BioLabs, Frankfurt, Germany) according to the manufacturer’s protocol. The Pyrosequencing Assay Design software version 1.0.6 (Biotage, Uppsala, Sweden) was used to derive rainbow trout-specific primers, ensuring that at least one primer from each pair spans an exon-exon junction. The selected set of genes and their corresponding primers are presented in Table 3. Multiplex quantitative real-time PCR (qPCR) was performed on 48.48 Gene Expression biochips, which were initially primed using the MX IFC Controller (Standard BioTools). Pre-amplified cDNA samples and primers were then loaded into the respective sample and assay inlets. Transcript levels were determined using the Biomark HD machine, following the manufacturer’s thermal protocol 'GE Fast 48 × 48 PCR + Melt v2.pcl’ (application type: gene expression; passive reference: ROX; assay: single probe). Raw Cq data were obtained using Fluidigm real-time PCR analysis software v3.0.2 (Standard BioTools), from which the relative expression of target genes was calculated using the ΔCt method. To reduce technical variation and account for differences in cDNA input, reverse transcription efficiency, and overall transcriptional activity, data were normalised using two suitable reference genes with expression stability indicated by coefficients of variation (CV) below 0.1. Normalisation factors were calculated as the geometric mean of the relative quantities of elongation factor 1-alpha 1(*eef1a1*)^49^ and ribosomal protein S5 (*rps5*)^50^. Relative expression levels of target genes were then normalised by multiplying their expression values by the corresponding normalisation factor.

**Table 3:** Sequences of primers used for quantitative real-time PCR analysis of gene expression in rainbow trout liver.

**Gene name**	**Gene product**	**Primer sequence 5’→3’** **(sense, antisense)**	**NCBI nucleotide acc.#**	**Amplicon length [bp]**
C3-3	Complement component C3, isoform 3	CGACCAGGGAAAGATGTTTGGA,	XM_021568201	168
		GTAGCCAAAATTAGCGCTGTACT		
Cat	Catalase	TGATGTCACACAGGTGCGTA,	XM_021557350	195
(LOC110486039)		CTCAACAACACTGAGCCCAC		
Cd36	CD36 antigen	TCTACTGATAGTGGGCATCGCA, TGAAGTGTTCTTAGCAGGGGGC	NM_001124511, XM_036934614	193
(LOC100136247)				
Crfb4 (il10rb)	Cytokine receptor family member b4 precursor	AGAGCACCGCTAAGGSCAAKG, CTGCTACAGGGTGGTCCGCT	NM_001281378, XM_036963669 XM_021578982	120
Gpx/	Glutathione peroxidase	CGAGCTCCATGAACGGTACG,	NM_001124525, HE687022, XR_002469241	183
LOC110494272		GTGGATGTGAACGGGAAGCA		
Hamp (LOC100135935)	Hepcidin	AGTGTTGCAGTTGCAGTGGTACTC,	XM_021595153	164
		GCGTCTGCCGGAGCATTT		
Ikba1	Inhibitor of nuclear factor kappa B alpha, isoform 1	AACCCTGGAGGAAAACAGTGAC, GAACAATCAGAGACAGACGGCG	NM_001124368	153
(LOC100136058)				
Ikba2 (LOC110497729)	Inhibitor of nuclear factor kappa B alpha, isoform 2	TGAAGTTGTCGCCAGTGAGCTC, AGAGCTGTCCTGCAATGAGCC	XM_021574049	187
Ikba3 (LOC110522049)	Inhibitor of nuclear factor kappa B alpha, isoform 3	AGAGTGGCCAATGTCGAAGTCT, GATAATATGTTACTGGACGCACAA	XM_021600117	175
Il4I1a	Interleukin 4 induced protein 1/L-amino-acid oxidase-like protein b	GAGACTATCTACTTTGAGGTGACA, AAACGTATCAGTCAGACTAGCAAT	XM_021567141	120
(LOC110492681)				
Mpo	Myeloperoxidase	GTGAAGGACCAGATCATTGTATTA,	DQ201133	152
		TAGTATTGCCTCTTTTAGGTGAGA		
Nfe2l2a	Nuclear factor erythroid 2-related factor 2, variant a	TTCCCACTGGTAGAGGCTACG,	XM_021597223	99
		GTCATGGCATGTGAGCTGCCA		
Saa5 (LOC118964931)	Serum amyloid A	GACATGTGGCGTGCATATGGC,	NM_001124436, XM_036980911, X99387	137
		CAGCAACAGTCATCAGTAATGG		
Serpine1 (LOC110531459)	Plasminogen activator inhibitor 1	GTCAACTGGTCCTGCCTAGGT,	XM_021614669, JQ801453	184
		GATTGAGGTGAACGAGGAGGG		
Sod1	Superoxide dismutase 1	TGCTTATGGAGACAACACCAAC,	XM_021590204, NM_001124329	156
		AATGTGGCTAAGATCAACATCCA		
Sod2	Superoxide dismutase 2	TCCCTGACCTGACCTACGAC,	XR_002474449	201
		GAGGTTTAATGGAGGAGGCC		

### Statistical analysis

All data were statistically evaluated using R (version R-4.4.3, 2025) statistical software. For the health parameter data, a mixed-effect model with the tank as a random factor was defined^51^. The corresponding residuals were checked for normal distribution and homoscedasticity or heteroscedasticity. Normalised gene expression levels are expressed relative to the mean of all samples and scaled by a factor of 1,000^52^. One-way analysis of variance (ANOVA) was conducted to evaluate blood, histology, and gene expression analysis data based on group measurements, followed by a Tukey multiple contrast test^53^. The historical data were evaluated using the Kruskal-Wallis test. A multi-factorial analysis of variance (two-way ANOVA) was used to evaluate the effects of diet, time, and their interaction on growth performance data, particularly FCR, SGR, FW, and WG. Statistical significance was determined using *P*-values of 0.05,0.01, and 0.001.

## Results

### Growth metrics

**Fig. 1:**
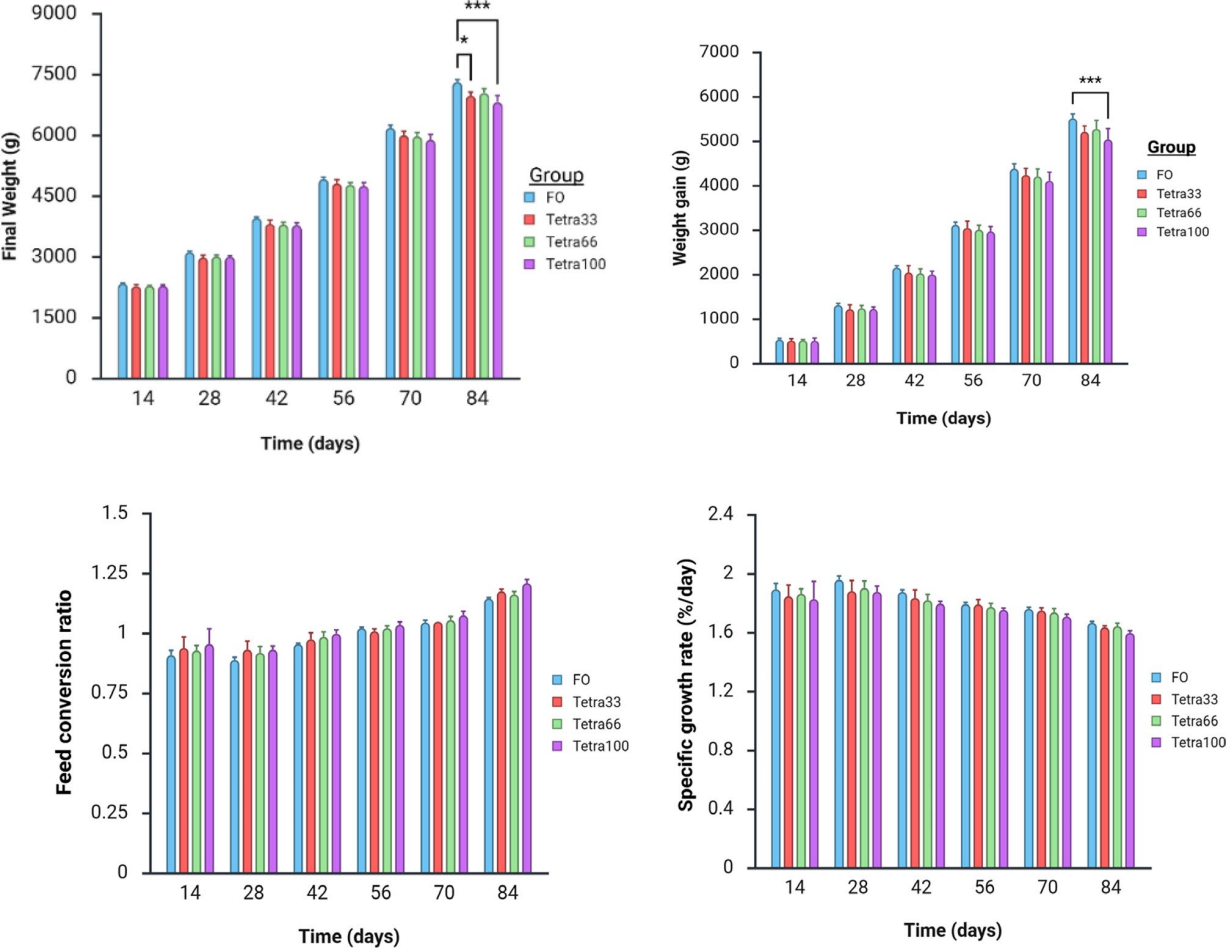
Growth parameters of rainbow trout (*Oncorhynchus mykiss*) (*n* = 20 per tank) fed diets containing *Tetraselmis chui* microalgae at 33% (Tetra33), 66% (Tetra66), and 100% (Tetra100) inclusion levels replacing fish oil. Final weight, weight gain, feed conversion, and specific growth rate data were recorded at 14^th^, 28^th^, 42^nd^, 56^th^, 70^th^, and 84^th^ day sampling points. A two-way Analysis of Variance (ANOVA) was performed to assess the effects of diet group vs sampling duration, and their interaction on the measured variables. Data are expressed in means + standard error (SE). Bars with asterisks denote significant difference at *P* = 0.001 (***), *P* = 0.01 (**), and *P* = 0.05 (*), while bars without an asterisk indicate no significant difference.

The present study evaluated the substitution of fish oil with Tetraselmis and its effects on selected growth indices (SGR, FCR, FW, WG), biochemical profiles, histological features, leucocyte counts and immune-antioxidant status in rainbow trout. Final weight remained statistically similar across groups from days 14 to 70. At day 84, the FO control (7321.65 ± 60.03 g) achieved significantly higher weights (*P* < 0.001) than Tetra33 (6984.70 ± 86.15 g) and Tetra100 (6823.93 ± 160.42 g), while remaining comparable to Tetra66 (7051.77 ± 107.30 g) (Fig.  1). Final weight also increased considerably in a time-dependent pattern (*P* < 0.001), whereas the interaction between diet and time was not significant. Weight gain did not differ significantly among groups between the 14- and 70-day sampling points. At day 84, the FO control (5519.65 ± 57.16 g) surpassed Tetra100 (5043.93 ± 142.09 g) but remained statistically comparable to that of the Tetra33 (5220.70 ± 73.95g) and Tetra66 (5281.77 ± 110.86g). Moreover, all experimental groups exhibited a pronounced time-dependent increase in weight gain (*P* < 0.001). In contrast, the diet × time interaction was not statistically significant (Fig.  1). FCR and SGR analysis revealed no significant dietary effect (*P* > 0.001) across the Tetra groups relative to the FO control. Regarding the sampling duration, a significant time-dependent increase in FCR and a corresponding decrease in SGR were observed (*P *< 0.001), whereas the diet × time interaction remained non-significant.

### Plasma chemistry

The present study evaluated the effects of replacing dietary fish oil with graded Tetraselmis inclusions on key biochemical indicators of juvenile rainbow trout (Table 4). Overall, the selected biochemical parameters, such as glucose, total protein, triglycerides, cholesterol, creatinine, lipase, amylase, bilirubin, and albumin, showed no significant differences (*P* > 0.05) among the Tetra groups compared with the FO control (Table 4). These parameters remained within the normal physiological range, which is important for promoting homeostatic functions in the fish. The liver enzyme activities (ALT, AST, and ALP) were also assessed to evaluate the potential impact on liver function (Table 4). Consistent with the biochemical profiles, enzyme activities in the Tetraselmis groups did not differ significantly (*P* > 0.05) from those of the FO control, indicating comparable ALT, AST, and ALP levels across the experimental groups.

**Table 4:** Biochemical profile of rainbow trout (*Oncorhynchus mykiss*) (*n* = 15 per group) fed diets containing *Tetraselmis chui* microalgae at 33% (Tetra33), 66% (Tetra66), and 100% (Tetra100) inclusion levels replacing fish oil. One-way analysis of variance (ANOVA) was used for statistical analysis. Data are expressed as mean ± standard error (SE). Values with an asterisk (*) denote significant differences; values without an asterisk show no significant difference at *P* < 0.05.

**Parameter**	**FO**	**Tetra33**	**Tetra66**	**Tetra100**	***P-value***
Glucose (mg/dl)	40.67 + 8.84	38.47 + 9.69	36.00 + 8.87	37.80 + 8.14	0.98
Total Protein (g/dI)	2.89 + 0.50	2.35 + 0.51	2.39 + 0.52	2.78 + 0.51	0.83
Triglyceride (mg/dI)	276.07 + 86.97	229.73 + 88.82	216.00 + 84.90	218.47 + 81.71	0.95
Alkaline phosphatase (U/I)	83.0 + 22.15	61.33 + 25.24	53.40 + 24.82	64.00 + 23.24	0.83
Alanine amino transferase (U/I)	14.73 + 2.36	17.60 + 3.12	14.33 + 2.94	16.67 + 4.25	0.84
Aspartate aminotransferase (U/I)	393.73 + 92.53	415.60 +102.37	295.46 + 88.62	364.93 + 89.03	0.81
Lipase (U/I)	32.93 + 0.62	32.33 + 0.80	33.00 + 0.72	32.53 + 1.06	0.91
Amylase (U/I)	583.33 + 107.85	763.87 + 108.41	421.60 +100.91	386.33 + 100.23	0.15
Cholesterol (mg/dI)	86.40 + 22.00	125.33 + 24.66	77.07 + 16.98	71.07 + 15.18	0.36
Tilirubin (mg/dI)	0.32 + 0.07	0.620 + 0.10	0.31 + 0.07	0.33 + 0.08	0.13
Albumin ((g/dI))	1.05 + 0.03	1.16 + 0.07	1.07 + 0.05	1.04 + 0.03	0.51
Creatinine (mg/dl)	0.68 + 0.37	0.77 + 0.39	0.86 + 0.43	0.73 + 0.38	0.99

### Leukocytes population

**Fig. 2:**
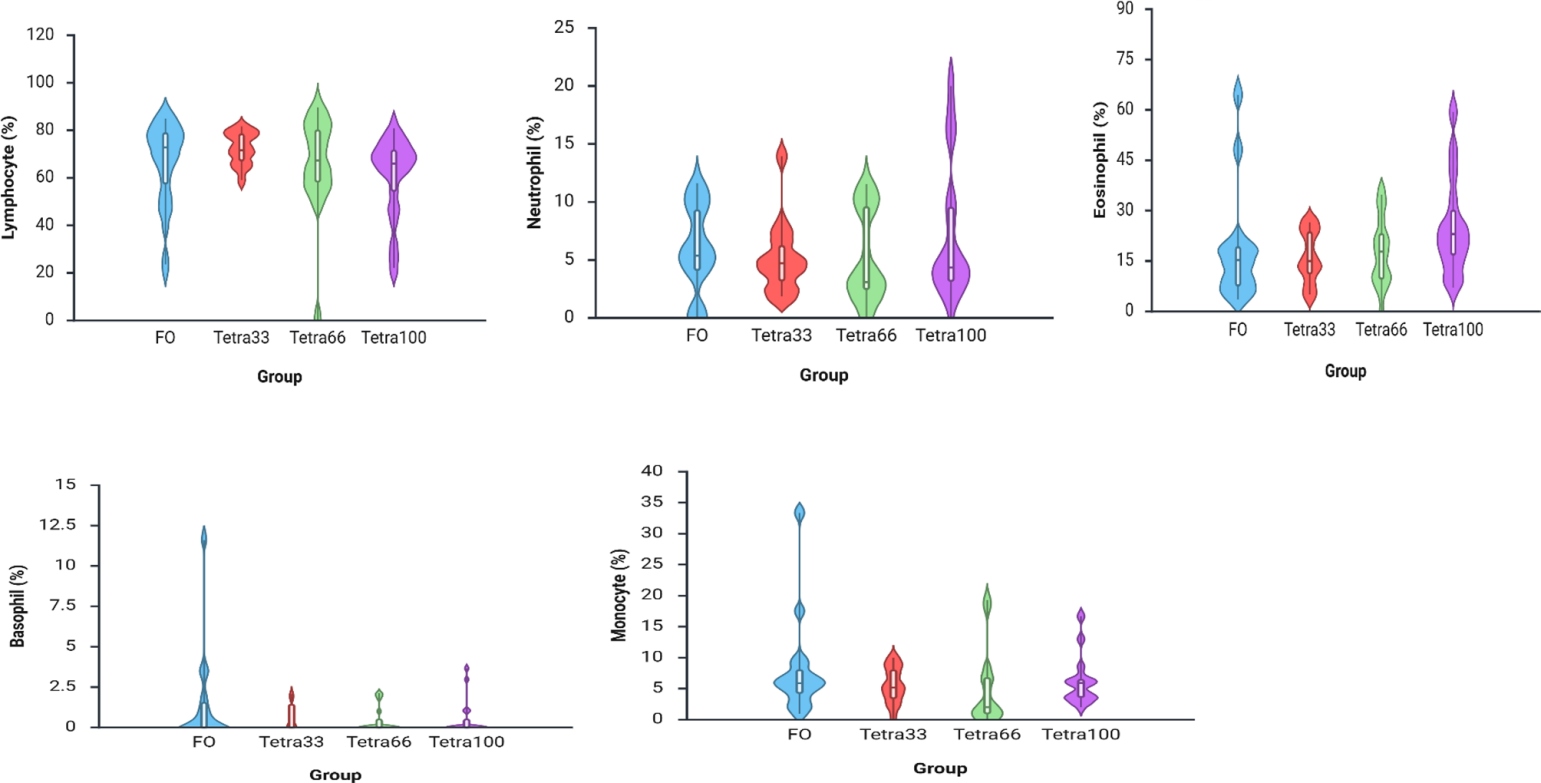
Leukogram of rainbow trout (*Oncorhynchus mykiss*) (*n* = 15 per group) fed diets containing *Tetraselmis chui* microalgae at 33% (Tetra33), 66% (Tetra66), and 100% (Tetra100) inclusion levels replacing fish oil (FO). One-way Analysis of Variance (ANOVA) was used for statistical analysis. Data are expressed in means + standard error (SE). Violin boxes with an asterisk (*) denote significant difference, while mean ball plots without an asterisk are not significantly different at *P* < 0.05.

The present study evaluated the impact of replacing fish oil with Tetraselmis on innate and adaptive immune markers, with a focus on leukocyte distribution in juvenile rainbow trout. Across all Tetra groups, the profiles of major leukocyte populations, notably neutrophils, basophils, lymphocytes, eosinophils, and monocytes, remained statistically comparable (*P* > 0.05) to those of the FO control (Fig.  2).

### Histological endpoints

#### Liver

**Fig. 3:**
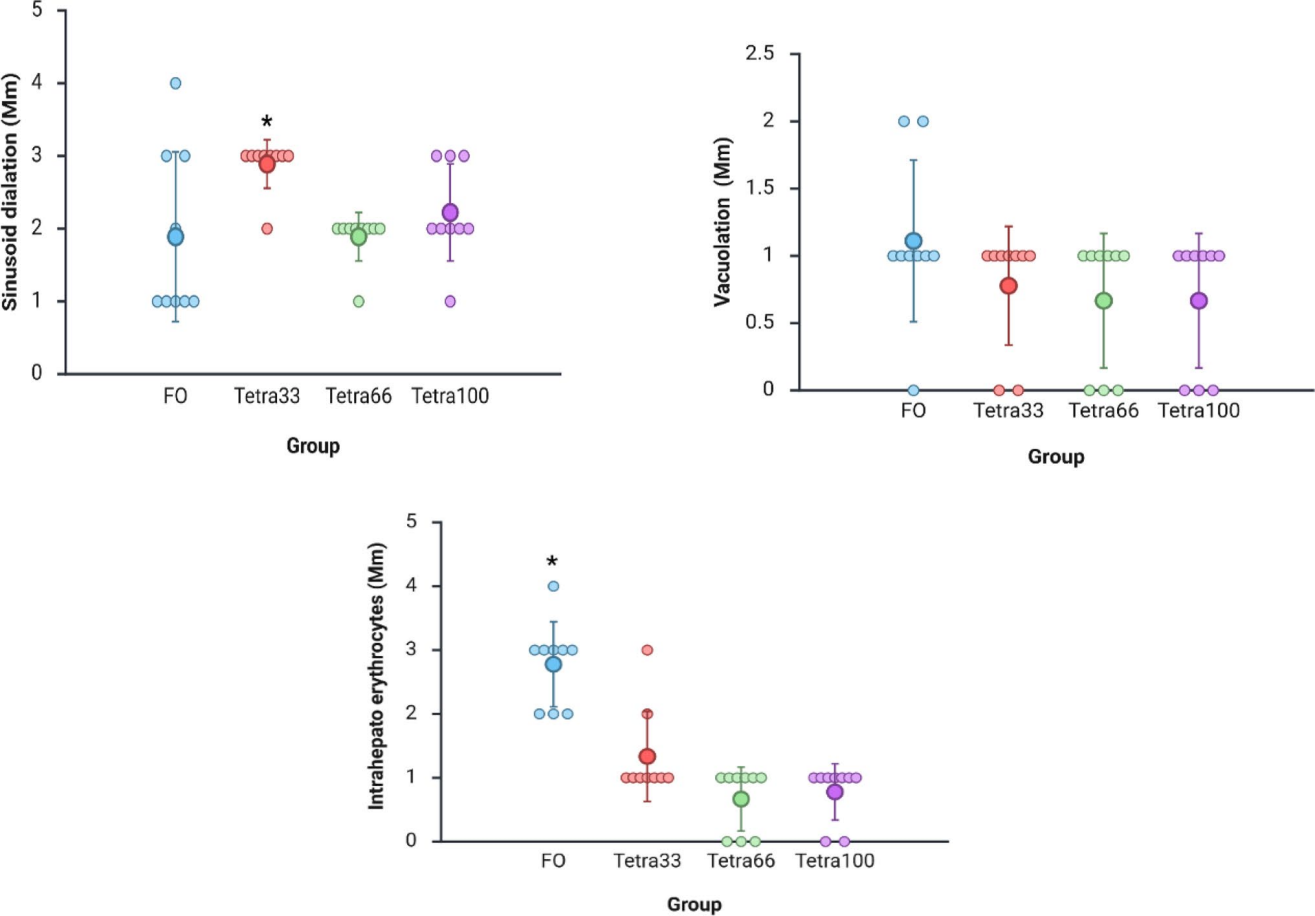
Liver histological parameters of rainbow trout (*Oncorhynchus mykiss*) (*n* = 9 per group) fed diets containing *Tetraselmis chui* microalgae at 33% (Tetra33), 66% (Tetra66), and 100% (Tetra100) inclusion levels replacing fish oil (FO). One-way Analysis of Variance (ANOVA) was used for statistical analysis. Data are presented as means ± standard error (SE). The ball plot represents the mean/median value. Ball plots marked with an asterisk (*) indicate significant differences, while those without an asterisk are not significantly different at *P* < 0.05.

**Plate 1:**
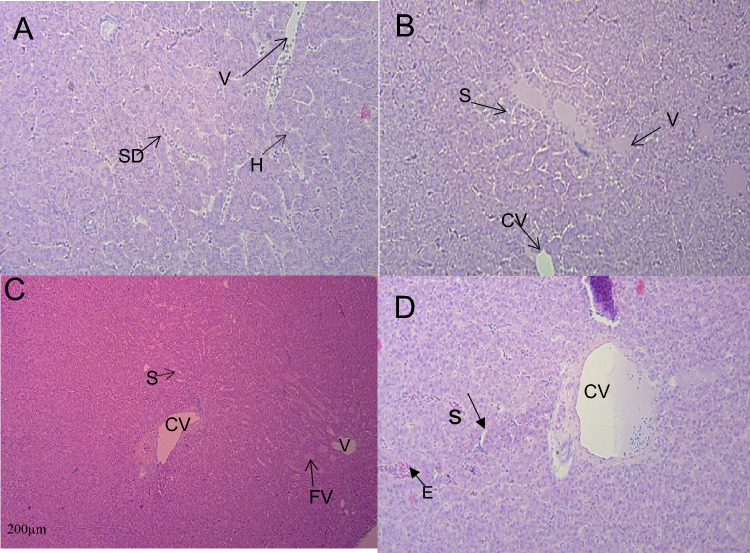
Representative liver photomicrograph section of rainbow trout (*Oncorhynchus mykiss*) (*n* = 9 per group) fed diets containing *Tetraselmis chui* microalgae at 33% (Tetra33), 66% (Tetra66), and 100% (Tetra100) inclusion levels replacing fish oil (FO). FO (A), tetra33 (B), tetra66 (C), tetra100 (D). Sinusoid (S), Sinusoid dialation (SD); Central Vein (CV), Vein (V), Cytoplasmic vacuolation (FV), Erythrocytes (E).

The present study assessed the impact of replacing dietary fish oil with graded Tetraselmis inclusions on liver and intestinal histomorphology in juvenile rainbow trout. Considering the histological examination of the experimental fish liver, the analysis identified consistent presence of the following histo-morphological parameters: sinusoid dilation, cytoplasmic vacuolation, and intrahepatic erythrocyte (Fig. 3, Plate 1). Quantitative severity scoring indicated that cytoplasmic vacuolization in hepatocytes of the Tetraselmis-fed fish was limited to normal-to-mild levels and was not significantly different (*P* > 0.05) from that observed in the FO control. Further, the FO group (2.80 ± 0.01) exhibited a significantly (*P* < 0.05) mild presence of intrahepatic erythrocytes compared with the Tetra33 (1.50 ± 0.10), Tetra66 (0.86 ± 0.00), and Tetra100 (0.95 ± 0.02) groups. In contrast, the Tetra33 group showed significantly (*P* < 0.05) mild-to-moderate dilated hepatic sinusoids relative to the Tetra66 (2.00 ± 0.01), Tetra100 (2.20 ± 0.02), and FO control (2.00 ± 0.00).

#### Intestine

**Fig. 4:**
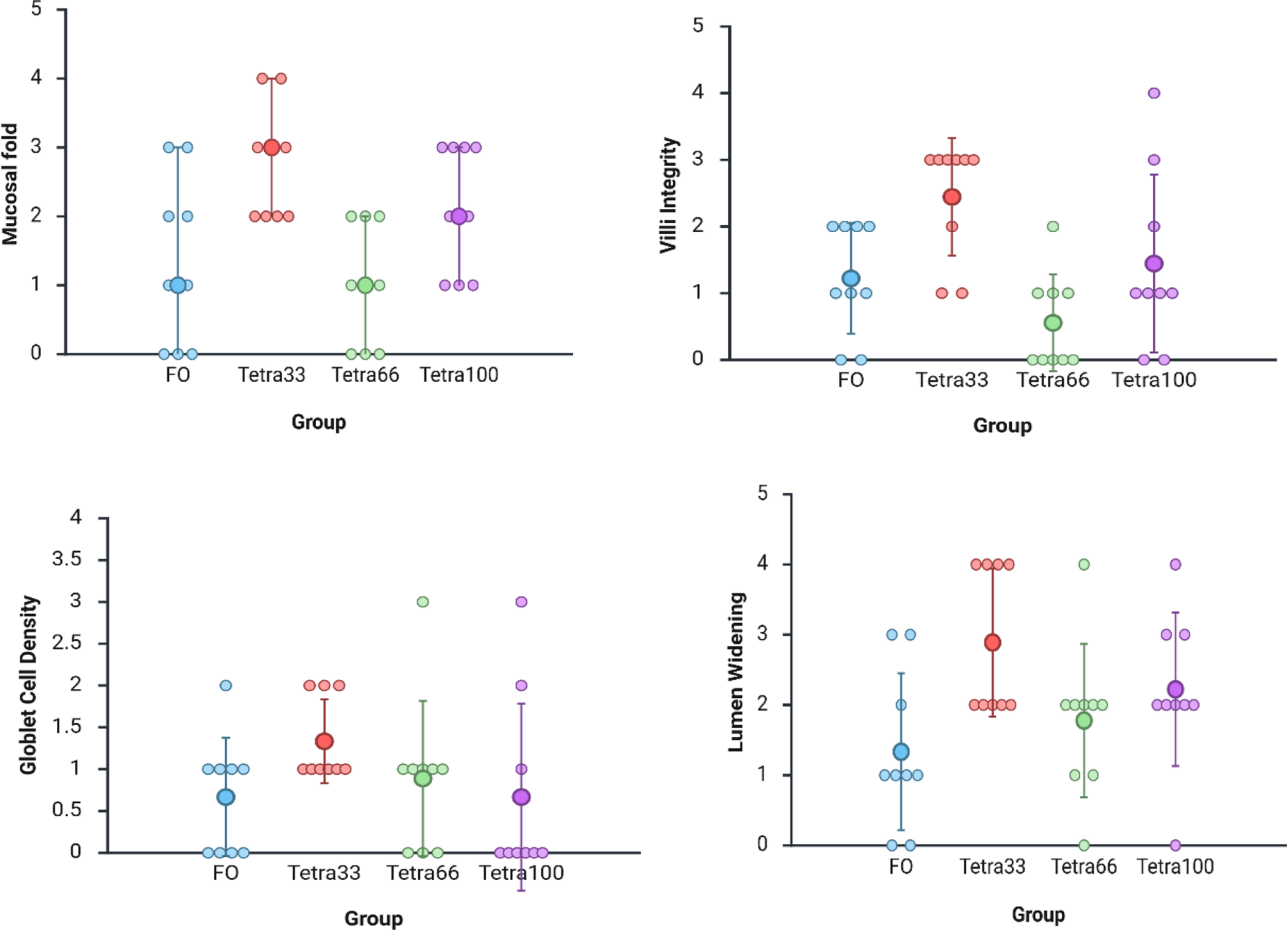
Intestinal histological parameters of rainbow trout (*Oncorhynchus mykiss*) (*n* = 9 per group) after 84 days on diets with graded inclusion levels of *Tetraselmis chui* at 33% (Tetra33), 66% (Tetra66), and 100% (Tetra100) replacing fish oil (FO). One-way Analysis of Variance (ANOVA) was used for statistical analysis. Data are presented as means ± standard error (SE). The ball plot represents the mean/median value. Ball plots marked with an asterisk (*) indicate significant differences, while those without an asterisk are not significantly different at *P* < 0.05.

**Plate 2:**
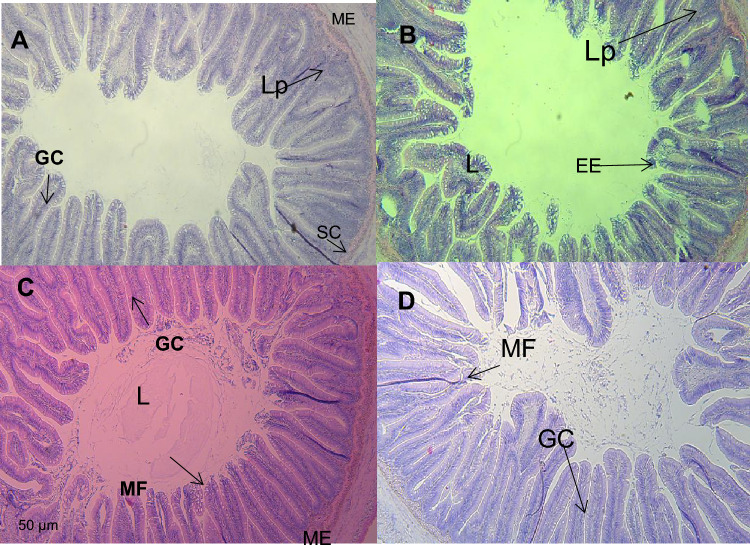
Representative intestinal photomicrograph section of rainbow trout (*Oncorhynchus mykiss*) (*n* = 9 per group) fed diets containing *Tetraselmis chui* microalgae at 33% (Tetra33), 66% (Tetra66), and 100% (Tetra100) inclusion levels replacing fish oil (FO). FO (A), Tetra33 (B), Tetra66 (C), Tetra100 (D). Mucosal fold (MF), Lumen (L), Globlet cell (GC), Eroded epithelium (EE), Lamina propria (LP), Stratum compactum (SC), Muscularis externa (ME).

Intestinal histology was analysed to assess the effects of replacing dietary fish oil with graded Tetraselmis inclusions on nutrient absorption, focusing on cellular and structural changes associated with digestive efficiency. The study findings revealed that varying dietary levels of Tetraselmis did not significantly affect goblet cell density, mucosal fold architecture, or lumen surface area (*P* > 0.05) in the intestines of the Tetra groups, compared to the FO control (Fig. 4, Plate 2), as indicated by non-significant mild-to-moderate histological measurements.

### Gene expression

#### Antioxidant-gene expression

**Fig. 5:**
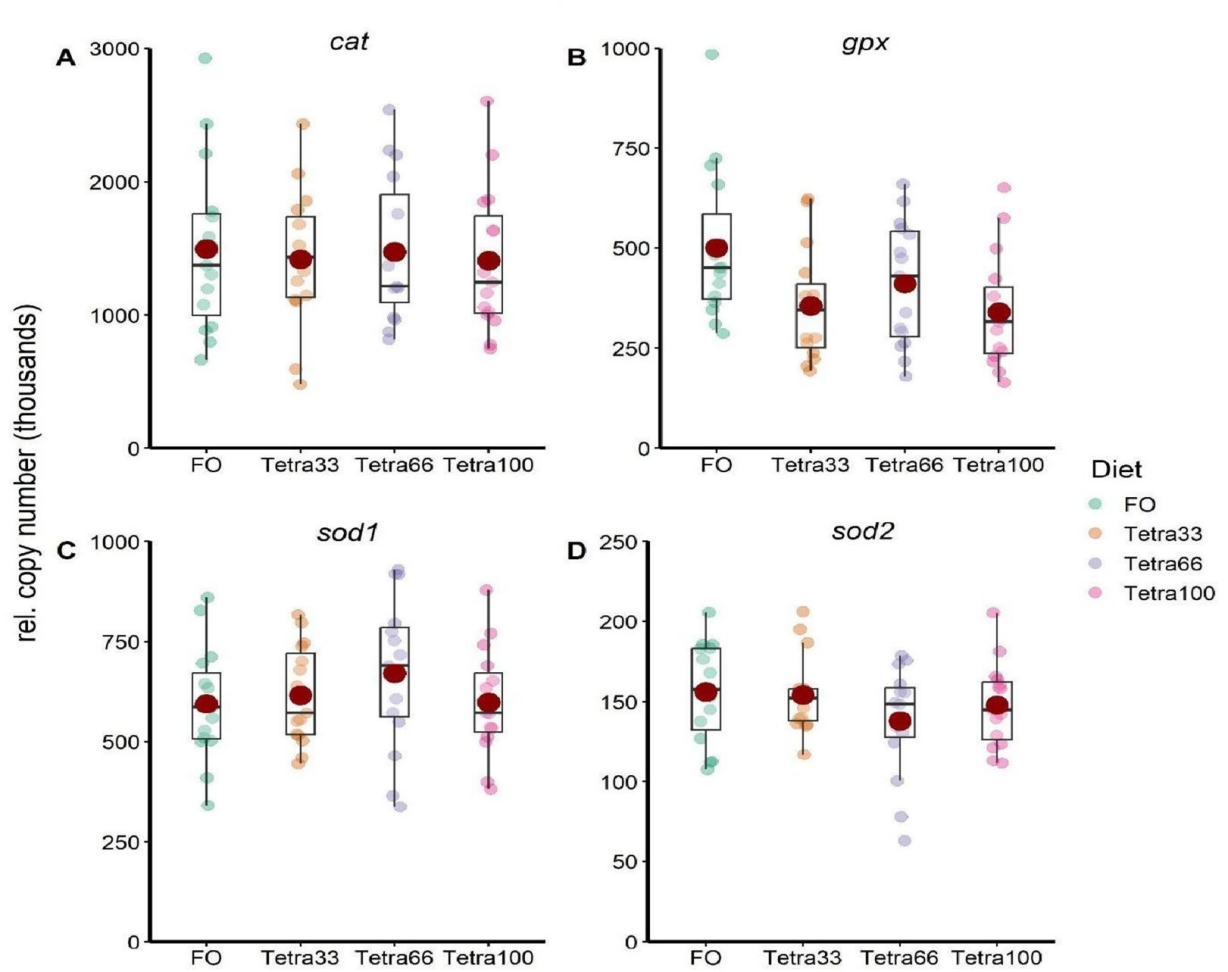
Antioxidant-gene of rainbow trout (*Oncorhynchus mykiss*) (*n* = 15 per group) fed diets containing *Tetraselmis chui* microalgae at 33% (Tetra33), 66% (Tetra66), and 100% (Tetra100) inclusion levels replacing fish oil (FO). The displayed transcripts encode for Catalase (*cat*), (b) Glutathione peroxidase (*gpx)*, (c, d ) Superoxide dismutase (*Sod1*and* Sod2*). Normalised gene expression levels are expressed relative to the mean of all samples and scaled by a factor of 1,000. One-way Analysis of Variance (ANOVA) was used for statistical analysis. The box plot represents the mean/median value. Ball plots marked with an asterisk (*) indicate significant differences, while those without an asterisk are not significantly different at *P* < 0.05.

The hepatic antioxidant gene-expression profile of juvenile rainbow trout was assessed to characterise the molecular responses elicited by replacing conventional fish oil with graded dietary inclusions of Tetraselmis. This analysis provides valuable insight into how *Tetraselmis* influences immune and stress-response pathways when traditional feed ingredients are replaced with alternative sources, such as microalgae. To further elucidate these effects, the study quantified the expression of key antioxidant-related genes, catalase (*cat*), glutathione peroxidase (*gpx*), and superoxide dismutase (*sod1 and sod2*) (Fig.  5), to characterise the oxidative status of the experimental fish. Relative expression of *cat*, *sod1*, *sod2*, and *gpx* did not differ significantly (*P* > 0.05) among Tetra groups compared with the FO control.

#### Immune-gene expression

**Fig. 6:**
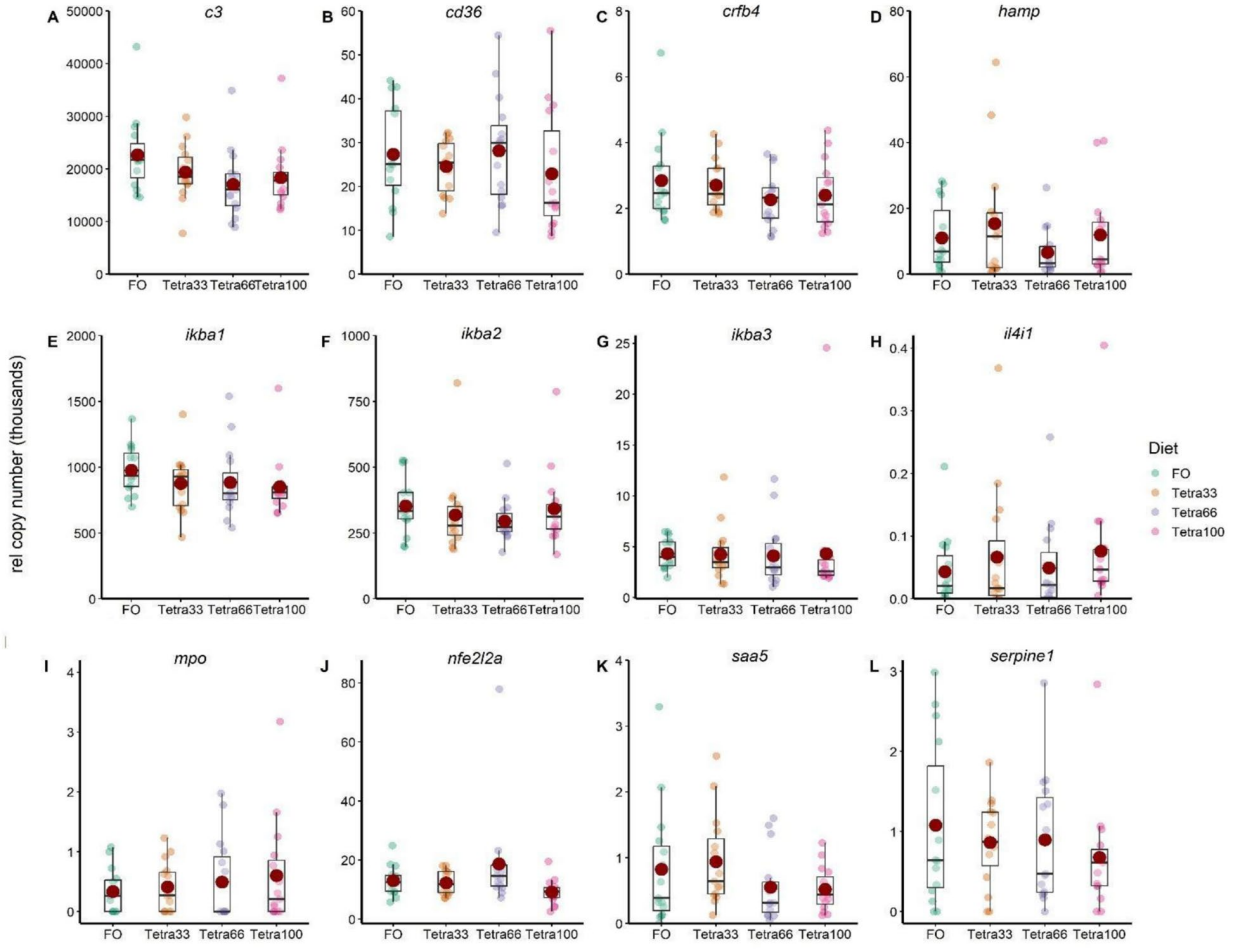
Expression of immune-related genes in rainbow trout (*Oncorhynchus mykiss*) (*n* = 15 per group) fed diets containing *Tetraselmis chui* microalgae at 33% (Tetra33), 66% (Tetra66), and 100% (Tetra100) inclusion levels replacing fish oil (FO). (**a**) Complement component 3 (*c3*), (**b**) Cluster of differentiation 3 (*cd3)*, (**c**) cytokine receptor family member b4 (*crfb4*), (d) hepcidin antimicrobial peptide (*hamp*), (**e**,**f**,**g**) nuclear factor of kappa light polypeptide gene enhancer in B-cells inhibitor alpha; *NFKBIA* (*ikba*), (**h**) Interleukin-4 (*il4i1*), (**i**) myeloperoxidase (*mpo*), (**j**) Nuclear factor erythroid 2-related factor 2 (*nfe212a*), (k) serum amyloid A 5 (*saa5*), (**l**) serpin peptidase inhibitor, clade E (nexin, plasminogen activator inhibitor type 1)(*serpine1*). Normalised gene expression levels are expressed relative to the mean of all samples and scaled by a factor of 1,000. One-way Analysis of Variance (ANOVA) was used for statistical analysis. The box plot represents the mean/median value. Ball plots marked with an asterisk (*) indicate significant differences, while those without an asterisk are not significantly different at *P* < 0.05.

Analysis of hepatic immune-gene expression revealed consistent patterns across the Tetra groups, with no significant deviations from the FO group (Fig. 6). The expression levels of complement component 3 (*c3*), hepcidin (*hamp*), cluster of differentiation 3 (*cd36*), cytokine receptor family B4 (*crfb4*), nuclear factor of kappa light polypeptides (i*kba1*, *ikba2*, *ikba3*), *interleukin* (*il4i1*), nuclear factor erythroid 2-related factor 2 (*nfe2l2a*), serum amyloid A protein (s*aa5*), serine protease inhibitor (*serpine1*), and myeloperoxidase (*mpo*), showed no significant variation (*P* > 0.05) across Tetra groups compared to the FO control, indicating that gene expression remained stable and within normal physiological bounds.

## Discussion

### Growth metrics

The use of alternative functional feed ingredients, including microalgae, in aquaculture diets presents several nutritional and health benefits for farmed species^4,13,54,55^. Within the framework of sustainable aquaculture, these ingredients serve as viable substitutes for costly and less accessible traditional feedstuffs. In this study, growth indicators, particularly final weight and weight gain trajectories, demonstrated that Tetraselmis-based diets did not compromise fish performance, with the highest growth observed in Tetra66 fish. Nonetheless, FCR values (0.9-1.2) across all groups were consistent with the normal range (0.8-2.0) reported for farmed rainbow trout^56^. SGR declined significantly over time, consistent with biological expectations in which growth potential peaks in the pre- and post-juvenile phases before gradually slowing as maturation progresses. This decline is typically driven by various biological factors, including metabolic demands and environmental conditions^57^. Detailed analysis of Tetraselmis’s dietary inclusion effect on rainbow trout’s growth and fatty acid metabolism can be found in the study of Simon et al.^13^. The comparable growth indices across the experimental groups underscore the nutritional potential and suitability of Tetraselmis (Table 2) to support fish growth, effectively serving as a substitute for fish oil^8,17,58^. These findings suggest that incorporating Tetraselmis into the diet supported feed efficiency and promoted effective nutrient utilisation^6,11^. Despite the lower DHA and EPA levels in the Tetra diets, growth performance was not compromised, underscoring the feasibility of incorporating functional ingredients, such as microalgae-derived products, as FMFO alternatives in aquafeeds. These findings highlight the potential of Tetraselmis to enhance the nutritional value of farmed fish without impairing growth. Furthermore, the maintenance of growth performance under reduced DHA and EPA intake can also be attributed to the fish’s endogenous biosynthetic capacity, whereby metabolic pathways utilise available precursors to generate these essential fatty acids^31,38,59^, effectively compensating for limited dietary provision. Sarker et al.^8^ reported a considerable increase in weight gain, SGR, and stable FCR in Nile tilapia fed a microalgae-blended diet (*Schizochytrium sp*) in replacement for fish oil and fish meal. Similarly, Carvalho et al.^25^ reported that gilthead sea bream (*Sparus aurata*) larvae fed a Schizochytrium (*Schizochytrium limacinum*) diet replacing fish oil exhibited improved growth and survival. According to Peng et al.^60^, supplementing dietary fish oil with Schizochytrium at 15g kg^-1^ improved growth (SGR, WG, FW) and feed utilisation (FCR) in Nile tilapia.

### Plasma chemistry

Plasma metabolites serve as key physiological indicators for assessing systemic metabolic and oxidative status of farmed fish^33,61^. The stability of glucose, cholesterol, amylase, lipase, and triglyceride levels indicates that energy reserves were adequately maintained to support metabolic function in the fish^33,62^. Similarly, normal serum protein and albumin concentrations reflect a well-regulated osmotic balance and efficient metabolite transport in the fish bloodstream^32,63^. The lack of significant variation in creatinine levels among the Tetra groups relative to the FO control further suggests stable biosynthesis in the blood, implying that renal function was not compromised^64^. The metabolic stability noted in fish may be linked to the PUFA composition of the diets (Table 2), particularly the contribution of EPA. EPA is extensively documented as a key regulator of metabolic processes in fish, supporting homeostasis and physiological function^65–67^. Katsoulis-Dimitriou et al.^68^ stated that microalgae (*Microchloropsis gaditana, Isochrysis sp.*, *Phaeodactylum tricornutum,* and *Schizochytrium sp.*) can serve as a viable substitute for fish oil, maintaining the metabolic capacity of fish, as observed in gilthead sea bream. The study findings align with Siddik et al.^55^^,^ who reported that fish effectively utilise microalgae-based lipids in various organs (liver, spleen, intestine, and kidney) to enhance metabolic activities and promote homeostasis. Habte-Tsion et al.^27^ reported that plasma albumin, amylase, total protein, and globulin levels remained unchanged in largemouth bass (*Micropterus salmoides*) fed a diet in which *Schizochytrium sp*. completely replaced fish oil.

Liver enzymes such as ALT, AST, and ALP serve as key biomarkers of liver function^33^. These results indicate that hepatic function was preserved, confirming that FO replacement with Tetraselmis inclusion did not impair liver performance. Furthermore, the absence of hepatotoxic effects underscores the nutritional safety of Tetraselmis, consistent with previous studies highlighting its antioxidant properties and favourable fatty acid composition^25,29,69^. Consistent with the present findings, Lee et al.^70^ reported that glutamic-oxaloacetic transaminase (GOT) and glutamic-pyruvic transaminase (GPT) levels were not significantly affected by the replacement of fish oil with *Schizochytrium sp*. in rainbow trout diets. Similarly, Habte-Tsion et al.^27^ found that ALP activity remained unchanged in largemouth bass fed a microalgae-based diet. Peng et al.^60^ reported that dietary replacement of fish oil with *Schizochytrium sp*. did not negatively affect liver enzyme activities in Nile tilapia.

### Leukocytes population

Leukocytes serve as essential indicators of innate immune function across diverse animal species, including fish. Primarily, these cells provide immune surveillance, protection, and modulate responses to different exposure scenarios^34,71,72^. This uniformity in circulating immune cell proportions indicates that dietary substitution with Tetraselmis did not perturb baseline immune homeostasis or trigger shifts typically associated with inflammatory activation. The absence of statistical deviations in both granulocytic (e.g. neutrophils, basophils, eosinophils) and agranulocytic (lymphocytes and monocytes) cell types suggests that the microalgal diets were immunologically well-tolerated and maintained normal leukocyte dynamics, reinforcing the view that Tetraselmis can replace fish oil without compromising innate immune competence (Siddik et al., 2023). This response may be attributed to the immunomodulatory properties of microalgal-derived PUFAs, which are known to regulate immune cell function and enhance immune defence in fish^69^. Relative to our findings, Sanchez et al.^73^ reported insignificant alterations in lymphocytes and neutrophil counts of Atlantic salmon fingerling fed diets enriched with *Nannochloropsis Gaditana* and *Schizochytrium sp.* microalgae. Later authors remarked that microalgae diets enhanced phagocytic activity in fish.

### Histological endpoints

#### Liver

Histomorphological evaluation of animal tissues provides biological endpoints that reflect exposure history and illuminate health outcomes, adaptive mechanisms, and exposure-driven interactions^35,74^. The liver plays a key role in detoxification and nutrient metabolism^75^. The absence of significant lipid deposition in hepatocytes suggests that dietary Tetraselmis may enhance hepatic function and promote fish health, following its rich essential fatty acid profile^6,29,76^. However, the moderately dilated sinusoids observed in fish fed Tetra33 suggest an enlargement of the liver capillaries, resulting in the leakage of erythrocytes into the liver. Hepatic sinusoids are specialised vascular channels within the liver, acting as critical pathways for the bidirectional transport of biomaterials. This enables the exchange of nutrients and oxygen with hepatocytes and supports the liver’s vital roles in detoxification and metabolism^71^. Nevertheless, the study findings suggest that Tetraselmis did not impair liver function, as evidenced by the unchanged activity levels of key liver enzymes (Table 4). Karapanagiotidis et al.^77^ reported that microalgae blend (*Schizochytrium sp*. and *Microchloropsis gaditana*) replacing fish oil did not markedly affect liver histomorphology, except for occasional instances of hydropic degeneration and haemorrhage. Serrano et al.^78^ reported decreased vacuolar degeneration of hepatocytes in rainbow trout fed a mixture of microalgae (*Schizochytrium limacinum* and *Nannochloropsis oceanica*) replacing fish oil. Neylan et al.^79^ also stated that microalgae diet had no negative influence on the liver tissue of sablefish (*Anoplopoma fimbria*).

#### Intestine

Histomorphological assessment of the intestine provides critical histological evidence of how experimental diets affect nutrient absorption, digestive integrity, and overall nutritional performance in aquaculture species. Nutrient absorption in fish is assessed histologically by examining villus morpho-architecture. Intestinal villi are finger-like epithelial projections that increase surface area for uptake and mediate transepithelial transfer of nutrients across the basolateral membrane into the circulation^35^. In the present study, mild histological alterations, such as disorganised villi arrangement and slight epithelial degradation, were observed in the villi of Tetra33 fish. These changes did not impair growth or absorptive function, indicating preserved intestinal function and suggesting that dietary Tetraselmis may exert immune-protective effects that limit intestinal inflammation^80^. Overall, no severe histological alterations were observed, indicating that Tetraselmis had no adverse effects on intestinal integrity or function in the fish, reinforcing the safety profile of Tetraselmis with respect to gut health^80^. Tetraselmis is reported to be rich in antioxidants, essential lipids and bioactive peptides^22,80,81^. Zhang et al.^29^ stated that black seabream (*Acanthopagrus schlegelii*) fed a microalgae diet exhibited normal villi density and arrangement, in contrast to fish on a high-fat diet.

#### Antioxidat-gene expression

Antioxidants play a vital role in protecting biosystems against oxidative damage caused by reactive oxygen species (ROS)^82^. Oxidative stress is an adverse health condition arising when the antioxidant system is overwhelmed by prooxidants, leading to the significant generation of ROS^83,84^. For farmed animals, this situation can arise from toxic exposures and stressful conditions, including suboptimal feeding stress, often induced by poorly formulated diets, particularly those with poor lipid content^29,85^. The lack of statistical variation in gene expression suggests that the antioxidant system was preserved and gene expression remained within normal physiological bounds, even under differing dietary inclusion levels of Tetraselmis replacing fish oil. Consequently, there was no evidence of antioxidant-system impairment or oxidative stress. Microalgae are a potent source of antioxidants vital for sustaining oxidative balance and enhancing the organism’s defence mechanisms^69,80^. Carvalho et al.^26^ reported that s*od* and *gpx* genes were not significantly altered in Meagre (*Argyrosomus regius*) fed a microalgae-enhanced diet replacing fish oil. A recent study indicates that black seabream exhibited enhanced antioxidant capacity when fed a diet containing a mixture of microalgae, compared to those fed with a high-fat diet^29^. This result suggests that dietary microalgae could confer health benefits and support physiological resilience in cultured fish. Similarly, Rosas et al.^28^ found that a partial replacement of fish oil with *Arthrospira platensis* microalgae in Mullet’s (*Mugil lisa*) diet increased the fish’s antioxidant response.

#### Immune*-gene expression*

The interplay between diet and host genomics is central to understanding how aquafeed ingredients modulate molecular pathways and immune function in cultured species, forming the basis of nutrigenomics in aquaculture^86^. Accordingly, this study examined the effects of replacing dietary fish oil with graded inclusions of Tetraselmis on hepatic immune-gene expression in rainbow trout. The study findings indicate that partial or complete replacement of fish oil with Tetraselmis did not elicit an inflammatory response. Consistent hepatic immune-gene expression across fish groups suggests maintained metabolic and immune homeostasis. The essential fatty acids in Tetraselmis (e.g., omega-3 and omega-6,Table 2) are functionally linked to enhanced immune responses and reduced inflammation in fish^87^**.** The observed immune stability correlated with unchanged myeloperoxidase (*Mpo*) activity and stable hepatic expression of immune-related genes *nfe212a* (*Nrf2*), *ikba*, *saa5*, *crfb4*, *il4i1*, and *cd36*, all of which are central to immune regulation and pathogen defence, suggesting preserved innate immune competence^88–94^. Serrano et al.^78^ observed no significant alteration in hepatic interleukin-12 (*il-12*) expression in rainbow trout fed diets in which fish oil was partially substituted with microalgae *(Schizochytrium limacinum* and *Nannochloropsis oceanica*)*.* Zhang et al.^29^ reported an enhanced expression of anti-inflammatory cytokine *interleukin-10*, while the expression of pro-inflammatory cytokine interleukin-1 beta was decreased in the liver of black seabream fed microalgae (*Phaeodactylum tricornutum, Tetraselmis sp.,Isochrysis galban*) supplemented diet.

Collectively, Tetraselmis diet provoked no adverse health challenge in the experimental fish, supporting its suitability as a fish oil alternative in rainbow trout nutrition, validating the study hypothesis. Microalgae diets have been reported to enhance antimicrobial and anti-inflammatory responses by modulating gene expression in aquaculture species^95,96^. Based on our findings, Tetraselmis show promise as a potential replacement for fish oil in aquaculture feeds^17,22^. Several studies point to their comparable nutritional profile to fish, providing high-quality protein, essential fatty acids, nutraceuticals, and a wealth of bioactive compounds, which could enhance feed nutrition and support sustainable aquaculture^6,11,76,97^.

## Conclusion

The findings of this study underscore the nutritional viability of Tetraselmis as a sustainable alternative to fish oil in aquafeeds for rainbow trout. Overall, replacing dietary fish oil with Tetraselmis preserved the experimental fish’s growth and health parameters, supporting its applicability and showing the greatest viability at a 66% inclusion rate. Significant variability in growth observed after the 70-day sampling point demonstrates that longer feeding trials are necessary. Such extended trials more accurately reflect the grow-out or later production phase and are therefore critical for determining the long-term impacts of alternative feedstuffs such as microalgae. Overall, these findings confirm that prolonged experimental durations are essential for robust evaluation of health, nutritional status, and sustainability outcomes. A deeper evaluation of Tetraselmis*’*s effects on gut microbiome composition and function, nutrient absorption dynamics, and biochemical pathways would clarify its nutritional efficacy and enable tailored feed formulations that optimise growth, immune resilience, and sustainability, supporting its integration into commercial aquafeeds. Although this study recorded relatively low levels of PUFAs (e.g. DHA and EPA) in Tetraselmis*,* further investigation of cultivation conditions and substrate selection is warranted.

## Data Availability

Data will be made available by the corresponding author (Stanley Iheanacho) upon reasonable request.
